# Empowering Women in Imaging Informatics: Confronting Imposter Syndrome, Addressing Microaggressions, and Striving for Work-Life Harmony

**DOI:** 10.1007/s10278-024-01285-6

**Published:** 2024-10-09

**Authors:** Mana Moassefi, Nikki Fennell, Mindy Yang, Jennifer B. Gunter, Teri M. Sippel Schmit, Tessa S. Cook

**Affiliations:** 1 Mayo Clinic, Rochester, MN USA; 2 Stanford Health Care, Stanford, CA USA; 3 SimonMed Imaging, Scottsdale, AZ USA; 4 Marquette University and Medical College of Wisconsin College of Engineering, Madison, WI USA; 5 Center for Practice Transformation in Radiology; 3-D and Advanced Imaging, Department of Radiology, University of Pennsylvania School of Medicine, 3400 Spruce St, 1 Silverstein, Philadelphia, PA 19104 USA

**Keywords:** Imaging informatics, Women in STEM, Gender equity, Diversity, Inclusion

## Abstract

For the past 6 years, the Society for Imaging Informatics in Medicine (SIIM) annual meeting has provided a forum for women in imaging informatics to discuss the unique challenges they face. These sessions have evolved into a platform for understanding, sharing experiences, and developing practical strategies. The 2023 session was organized into three focus groups devoted to discussing imposter syndrome, workplace microaggressions, and work-life balance. This paper summarizes these discussions and highlights the significant themes and narratives that emerged. We aim to contribute to the larger conversation on gender equity in the informatics field, emphasizing the importance of understanding and addressing the challenges faced by women in informatics. By documenting these sessions, we seek to inspire actionable change towards a more inclusive and equitable future for everyone in imaging informatics.

## Introduction

The unique challenges facing women in imaging informatics in medicine (WIIM) are often insufficiently discussed and addressed in the professional setting. For the past several years at the Society for Imaging Informatics in Medicine (SIIM) annual meeting, we have tried to shed light on these critical issues by hosting sessions focused on women in informatics. These sessions began as a forum for sharing ideas and quickly became a movement toward understanding and change. Led by various women leaders in imaging informatics, each employed in different work settings, these events have created a space where voices could be heard, experiences shared, and strategies developed. Participation in the WIIM session is open to all SIIM members, without restrictions based on gender or any other related criteria. This paper aims to provide an overview of the key points and discussions from our recent sessions, emphasizing the most recent one held in Austin, TX, USA, at the 2023 Annual Meeting.

## Session Formats and Topics

The WIIM sessions at SIIM have taken different forms since the first session in 2017 (Table 1). Although sessions have typically consisted of small group discussions with report-outs, discussion themes have not always been assigned. In some cases, groups chose their discussion topic, while in others, topics were pre-defined. In 2022, there were three assigned topics and a fourth “people’s choice” topic voted on by session attendees. In 2023, the moderators recommended three topics: imposter syndrome, microaggressions in the workplace, and work-life balance.

**Table 1:** Format of the different WIIM sessions held at SIIM. Our database does not contain the number of participants for the year 2017

Year	Format	Number of participants
2017	Moderated small group discussion with report-outs	N/A
2018	#HeforShe panel discussion questions and answers	70
2019	Interactive roundtable session discussing microaggressions, unconscious bias, and conflict resolution	60
2020	Didactic lectures with questions and answers (virtual)	23
2021	Panel discussion (virtual)	61
2022	Moderated small group discussion (4 groups) with report-outs	23
2023	Moderated small group discussion (3 groups) with report-outs	65

These themes were chosen for their relevance and impact on women’s professional journeys in the field of informatics. The general attendance for the sessions remained relatively consistent, with the exception of two years. In 2020, there was a notable decrease likely due to the COVID pandemic and the virtual format. The 2022 meeting represented the first in-person meeting post-COVID, and there was no virtual participation option for this session. The overall participation largely depended on the timing and location of other concurrent sessions. Session participants selected the discussion group they wished to join. Participants engaged in one of the discussion topics in each group, sharing personal experiences and insights. This exchange was not just about expressing concerns but was a collective effort to identify and articulate the subtle yet significant barriers that women face in informatics. The discussion groups were facilitated by two moderators who documented the contributions made by group members. In the initial round, each member of the group discussion contributed one key point about the topic, which the moderators documented. The remainder of the discussion was conducted voluntarily by the members, and their ideas were collected accordingly. Upon conclusion of the sessions, a comprehensive summary of these points, supplemented by additional insights agreed upon by the organizing team, was compiled and subsequently reported in this paper.

### Imposter Syndrome

Imposter syndrome, a term first explained in the 1970s, refers to the internal experience of believing that one is not as competent as others perceive one to be [1]. Despite external evidence of their competence, individuals with imposter syndrome remain convinced that they are frauds and do not deserve the success they have achieved. This psychological pattern may be particularly prevalent in the field of informatics, a domain where rapid advancements and high-skill expectations can exacerbate feelings of self-doubt and inadequacy. Women in informatics often face unique challenges in this historically male-dominated environment, which can further fuel these feelings [2, 3].

Context plays a vital role in imposter syndrome as individuals look to others as role models to identify characteristics of an authentic academic or professional. In comparing themselves to those they deem to be leaders in their field, women may notice differences and begin to feel like counterfeits. Deeply embedded in the dynamics of privilege and oppression, such phenomena initiate feelings of being an outsider, reinforcing the prevailing dominant narrative and manifesting as a sense of not belonging [4]. The theory of imposter syndrome has been researched, but few practical solutions have been developed [5, 6]. Although feelings of self-doubt can originate internally, a study reveals that these feelings are more likely tied to the institutional or systemic discourses prevalent in academic environments. This observation is crucial as it uncovers the covert institutional and systemic structures that foster the imposter phenomenon [7]. Crawford and colleagues discovered a notable link between impostor syndrome and community college employees’ self-reported difficulty in managing work-life balance. Interestingly, this correlation diminished when employees felt a stronger sense of support from their organization. This finding points to the important role that managers and executives can play in lessening the impact of impostor syndrome on their staff [8].

### Workplace Microaggressions

Microaggressions in the workplace, particularly those experienced by women, are a concerning aspect of modern professional life. Microaggressions are subtle everyday behaviors, whether in spoken words or nonverbal cues, stemming from unconscious biases, concealed prejudices, or underlying hostility [9]. Men are often stereotypically perceived as competent and assertive [10], qualities that align with the competitive nature of the informatics field. In contrast, women are typically viewed as warm and nurturing [10]. Such stereotypes influence how women in academia are evaluated, with assessments often focusing on their appearance and personality—expecting them to be nurturing and empathetic—rather than on their professional skills [11, 12]. Women who do not conform to these expectations may face harsh judgment and microaggressions. These subtle acts of discrimination can create an unwelcoming and hostile atmosphere for women, particularly in male-dominated fields such as informatics and other similar environments [10].

Encountering an increased frequency of microaggressions is linked to higher levels of depression, anxiety, and a sense of not belonging in the workplace [13]. Women who frequently encounter microaggressions are more likely to leave their jobs, contributing to the gender gap in specific industries [14]. This loss of talent has a negative impact on workplace diversity and productivity [15, 16]. Research consistently demonstrates that diverse teams are more productive and innovative [17, 18]. However, according to data from the UNESCO Institute for Statistics in 2019, women make up only 29.3% of the global research and development (R&D) workforce [19].

### Work-Life Balance

Work-life balance is defined as the degree to which individuals maintain equal engagement and satisfaction in their work and personal roles. Work-life balance presents significant challenges for women in any profession, including the dynamic and demanding field of informatics. The complexities of this balance are influenced by many factors, including personal passions, self-care priorities, cultural upbringing, career choices, and societal stereotypes. The high-paced and technology-driven nature of the informatics field often magnifies these challenges. Practices promoting work-life balance significantly enhance the quality of professional and personal life among employed women [20–23]. This improvement is notably influenced by their psychological characteristics and attitudes [24–26]. Understanding and addressing these issues is crucial for women’s well-being and professional success in this field.

This framework can be extended to other underrepresented minorities, such as ethnic and racial minorities, LGBTQIA + individuals, and people with disabilities, who also navigate similar barriers in the field of informatics [27–31].

## Results

In the 2023 WIIM session, approximately 62 women and three men participated in the session. Here, we share a summary of the discussion points held by each group on their chosen theme. No identifiable information is included that might unfairly spotlight any individual discussant. Rather, we present broad discussion details as summarized by the group moderators.

### Imposter Syndrome

The group discussing imposter syndrome tackled three aspects: factors leading to imposter syndrome, the role of workplace culture, and coping strategies.

Imposter syndrome is not solely an internal struggle; external factors, especially workplace culture, often influence it. A critical factor that promotes imposter syndrome is the reluctance to share one’s feelings and experiences with others. Many individuals with imposter syndrome keep their doubts and insecurities hidden, exacerbating their internal struggles. Moreover, working in a competitive culture with limited opportunities can worsen feelings of inadequacy and fear of not measuring up to one’s peers. Addressing these external factors is essential in combating imposter syndrome. One approach is to make mental health tools and resources available discreetly and pervasively within the workplace, encouraging employees to seek help when needed. Leadership within an organization plays a pivotal role in mitigating imposter syndrome. Strong and supportive management sets the tone for a workplace culture that recognizes and values every individual’s contributions, ultimately reducing the prevalence and impact of imposter syndrome among employees. This discussion conclusions are consistent with the information found in the literature [8, 32, 33].

**Fig. 1:**
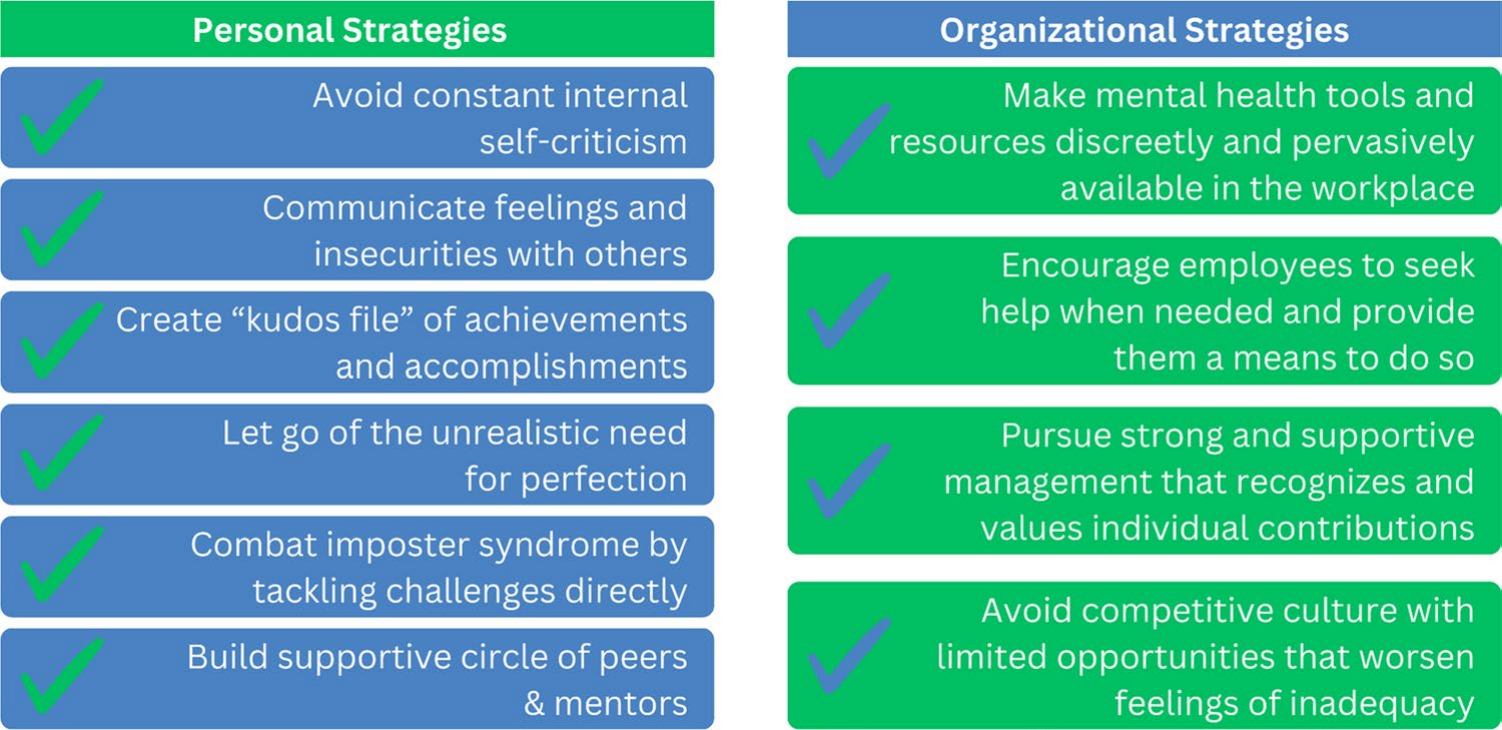
A summary of reviewed strategies for overcoming imposter syndrome in the workplace

Those who experience imposter syndrome often find themselves trapped in a cycle of self-doubt and insecurity. Various coping strategies exist but are rarely taught and infrequently discussed in the professional setting. One of the most effective approaches is to seek external validation and avoid constant self-criticism. Some individuals tend to compensate for their perceived inadequacies by working excessively hard. While this may offer temporary relief, it can harm mental well-being. Creating a “kudos file” to document achievements and reminding oneself of these accomplishments, particularly during performance reviews, can help combat the negative self-talk associated with imposter syndrome. Moreover, letting go of the unrealistic need for perfection is crucial, as it is an unattainable goal that perpetuates feelings of inadequacy. Building a supportive circle of peers and mentors who encourage personal growth and pushing oneself to be better can also be helpful (Fig. 1).

### Workplace Microaggressions

Participants in this small group discussion were asked to share a workplace microaggression experience. They reported experiencing stereotypes, supervision by male peers, double standards, and pay disparities.

Participants shared their experiences facing microaggressions through stereotypes that undermine their capabilities. Some reported being frequently interrupted or talked over during meetings, leading to their ideas being overlooked or attributed to male colleagues. Others mentioned instances where ideas presented by female colleagues in emails or during meetings ended up being incorporated into a male colleague’s project or work without attribution or acknowledgment. Many participants felt compelled to exert extra effort in their work, even in the face of physical challenges, to avoid such potential microaggressions in the future. One participant recounted an experience of a transgender colleague who encountered significantly fewer instances of being asked to explain or justify their actions in the workplace as a male than they had as a female. Previous research on gender transition and salary details has demonstrated that the incomes of transgender individuals are influenced by their transition status and whether they have disclosed their gender identity [34].

Several participants, primarily women working on hardware, reported being asked to be supervised by a male colleague, even though their male peers did not face similar requests. Women also reported microaggressions when held to standards different from those of their male counterparts. This included examples of male colleagues or supervisors commenting about their appearance, which diverted attention from their professional contributions.

There was a similar experience among the discussion group, indicating that microaggressions were also found to exacerbate gender pay disparities. Women may not negotiate for higher salaries or promotions due to the fear of being perceived as overly aggressive, leading to earning less than their male counterparts over time.

Many suggestions were made during the discussion on how to enhance the workplace environment to decrease microaggressions. These included conducting dedicated educational sessions and encouraging women to share their experiences during these sessions, thereby raising awareness among men about unconscious biases and inadvertent aggressions. The discussion also focused on advocating for each woman to allocate time to educate their families about potential biases and the manifestation of microaggressions.

### Work-Life Balance

Stereotypes, particularly those about millennials and elder millennials, can create additional pressure in the workplace. These groups often feel compelled to prove their capabilities to a greater extent, leading to self-induced stress. This need to prove themselves disrupts their work-life balance, as they devote more time and energy to work, often at the expense of personal life and well-being.

**Fig. 2:**
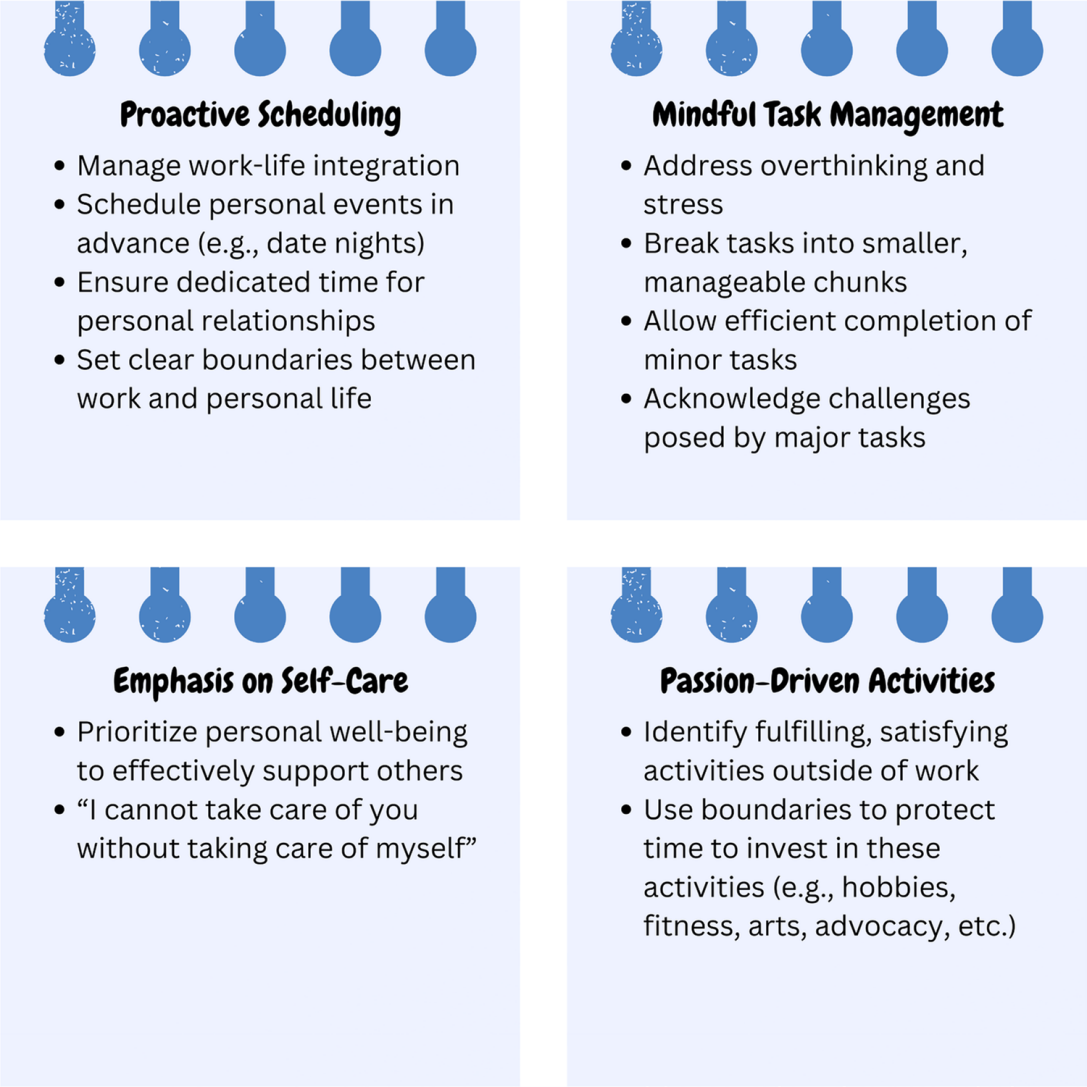
Selected strategies for enhancing work-life balance

Seeking support from spouses, partners, or close relatives and expressing gratitude for their assistance and presence was mentioned as a strategy towards work-life balance. Additional recommendations and experiences to effectively manage and overcome the mental stress associated with work-life balance included the following:

- One participant highlighted the importance of self-care, emphasizing that taking care of oneself is essential for being able to help others. This was concisely stated as “I cannot take care of you without taking care of myself.”
- In the pursuit of work-life balance, a practical approach is to engage in activities other than work and personal responsibilities that are both fulfilling and passion-driven. This was exemplified by a participant who found balance by becoming a gym instructor, a role providing not only financial benefits but also personal satisfaction and meaning. Integrating activities that she loved and found meaningful into her routine helped her achieve more vital work-life harmony.
- Another effective method for managing work-life balance stress involves proactive scheduling and mindful compartmentalization of tasks. By placing events, such as date nights or quality time with spouses, on the calendar well in advance, individuals can have better control over their time before it becomes consumed by other commitments. This approach not only ensures dedicated time for personal relationships but also helps set boundaries between work and personal life. Furthermore, expecting too much of oneself can lead to overthinking and stress; therefore, being more mindful and dividing life into manageable segments or “packets” can help with this pressure. Tackling tasks in smaller chunks allows for the efficient completion of minor tasks while acknowledging that larger items may present more significant challenges. This strategy of breaking down tasks and giving specific times for different aspects of life, such as career, personal time, and fitness, helps create a more balanced and manageable routine (Fig. 2).

## Conclusions

The discussions and insights shared during these WIIM sessions at SIIM underscore the ongoing need for open dialogue and collective action to address women’s unique challenges in the informatics field. From confronting the insidious effects of imposter syndrome to navigating the subtle yet damaging impacts of workplace microaggressions and striving to achieve a harmonious work-life balance, the narratives and experiences shared by participants serve as a powerful reminder of the barriers that still exist and the work that remains to be done. In this session, we addressed the challenges related to gender minority issues faced by women. However, research indicates that similar challenges are encountered by all minority and marginalized groups, encompassing demographic factors such as race or ethnicity, gender, sexual orientation, and age [27–31]. Future efforts should involve providing similar forums and conducting comprehensive studies in the field to further explore and address these topics. As we look ahead, the WIIM initiative remains steadfast in its commitment to supporting a more inclusive and equitable environment for all within the informatics community.

## Abbreviations

WIIM: Women in imaging informatics in medicine
SIIM: Society for Imaging Informatics in Medicine
